# The intersection of TB and health financing: defining needs and opportunities

**DOI:** 10.5588/ijtldopen.24.0324

**Published:** 2024-09-01

**Authors:** W.A. Wells, S. Waseem, S. Scheening

**Affiliations:** ^1^ US Agency for International Development, Washington, DC, USA;; ^2^ Credence Management Solutions, Vienna, VA, USA;; ^3^ Management Sciences for Health, Arlington, VA, USA;; ^4^ Open Development, Washington, DC, USA.

**Keywords:** tuberculosis, domestic resource mobilization, strategic purchasing, national TB programs, NTPs

## Abstract

TB is an airborne public health threat, so the reponse to TB has been defined mainly through the lens of vertical, public-sector national TB programs (NTPs). However, TB exists within a broader health systems and health financing context. Here, we examine the intersection between the particular needs of TB programs and the broader health financing landscape. This includes the strategies needed to finance both the clinical and public health aspects of the TB response. In high-burden countries, the resource mobilization and strategic purchasing approaches described here will be critical if we are to maximize the reach and impact of the TB response.

## RÉSUMÉ

La TB est une menace de santé publique transmise par l'air, de sorte que la réponse à la TB a été définie principalement à travers le prisme des programmes nationaux de lutte contre la tuberculose (NTP), verticaux et relevant du secteur public. Cependant, la TB s'inscrit dans un contexte plus large de systèmes de santé et de financement de la santé. Nous examinons ici l'intersection entre les besoins particuliers des NTP et le paysage plus large du financement de la santé. Il s'agit notamment des stratégies nécessaires pour financer les aspects cliniques et de santé publique de la lutte contre la TB. Dans les pays à forte charge de morbidité, les approches de mobilisation des ressources et d'achat stratégique décrites ici seront essentielles si nous voulons maximiser la portée et l'impact de la lutte contre la TB.

In 2022, TB was the second leading cause of global deaths from an infectious disease after COVID-19, and caused almost twice as many deaths as HIV.^1^ The financing needs of TB programs in high-burden countries have significantly increased in recent decades. This is driven by improvements in available tools and a recognition of the need for more robust efforts, particularly in areas such as TB prevention.^2,3^ Throughout this period, donor funding has been an important contributor to TB outcomes, and in high TB burden countries the proportion of funding from donors is higher for TB than for health overall (Table 1).^4^ However, since 2013, donor funding for TB in low- and middle-income countries (LMICs) has been largely static at approximately US$ 1.1 billion per year.^1^ Domestic financing accounts for 39–94% of overall TB program costs (Table 2),^1^ and is the most promising opportunity for growth in TB financing in the future.^4^ Considerable advocacy has been devoted to communicating this broad message. However, there has been relatively little focus on the detailed technical approaches needed to mobilize and effectively use domestic financing to provide a more robust TB response.

**Table 1.** Donor and domestic funding for health and TB in selected higher-income, high TB burden countries.

Country	Proportion of external funding out of domestic + external: health (A)^*^	Proportion of external funding out of domestic + external: TB (B)^†^	B/A	B-A
	%	%		
Ukraine	1.3	42	31	41%
Philippines	1.5	45	29	44%
Indonesia	3.6	41	11	38%
Vietnam	8.2	85	9.4	77%
India	6.1	34	4.6	28%
Kyrgyz Republic	9.9	39	2.9	29%

* Calculated from the WHO Global Health Observatory (https://www.who.int/data/gho/data/indicators) using 2021 figures for External Health Expenditure (EHE) and domestic general government health expenditure (GGHE-D).

† Calculated from the Global Tuberculosis Programme (https://www.who.int/teams/global-tuberculosis-programme/data) using 2021 figures.

**Table 2.** Proportion of TB financing from domestic sources in 2022.

Country category	Proportion of TB financing from domestic sources
All low- and middle-income countries (LMICs)	81% (US$4.7 billion out of US$5.8 billion)
Brazil, the Russian Federation, India, China and South Africa (BRICS)	94%
Outside BRICS, the 26 high TB burden and two global TB watchlist countries (Cambodia and Zimbabwe)	48%
Low-income countries	39%

Financing is just one of the health system building blocks,^5^ but it impacts the entire operation of the health system. The way that financing is organized can shape the governance approach, human resource planning, the demands for and use of health information, responsibilities for commodities, and the extent to which services are coordinated.^6^ The way that financing is organized can also be a major driver of health system outcomes such as equity, quality and resource optimization.^7^

The TB and health financing communities do not often find opportunities to mix and exchange views. Here, we seek to define opportunities for health financing reforms that would improve outcomes in high TB burden countries. This effort will require a critical mass of country-based leaders and implementers who are motivated, able and willing to work at the intersection of health financing and TB. We do not focus in-depth on technical efficiency (i.e., choosing the most efficient way to implement a given TB implementation task) or allocative efficiency (i.e., choosing the TB activities that will have the most impact), as these two topics focus more on TB programmatic choices rather than health financing considerations. Instead, we focus on the particular characteristics of TB programs that require a specific health financing approach and then describe how those characteristics play out: first in TB domestic resource mobilization, followed by strategic purchasing for TB. The application of these concepts should result in an increased amount of resources for TB and greater efficiency in the use of those resources.

## THE ARGUMENT FOR A TB-SPECIFIC APPROACH TO HEALTH FINANCING

As an airborne infectious disease, TB is a public health threat, and the response to TB requires multiple public health actions (PHAs) to contain its spread. We define PHAs as the actions that primarily address the public health concerns arising from TB, rather than acute clinical concerns of the patient. The latter is addressed by what we define as individual health actions (IHAs), i.e., the clinical steps of diagnosis and treatment. The importance of PHAs and the public health nature of the TB response requires a specific health financing approach (Table 3). As an example of the importance of PHAs, if a health system treats TB patients who self-present in public facilities but takes no other steps, it will miss most people in need of care. In high TB burden countries, up to two-thirds of TB patients found in prevalence surveys had not previously sought care in formal facilities.^8^ In addition, up to half of those who do seek care may be missed because facilities do not conduct intensified case finding by actively screening for TB.^8^ Left unaddressed, these case-finding gaps lead to ongoing TB transmission and increased morbidity and mortality.

By contrast, when proactive efforts are made, TB case finding can be almost doubled by private provider engagement,^9^ or almost tripled by active case-finding initiatives in the community.^10^ Pro-active screening in other specific settings, such as mines and prisons, also plays a critical role in TB programs,^11^ which is why multisectoral planning and budgeting is so important.^12^

Additional PHAs go beyond these important case-finding efforts. Because patients may feel better after 2–3 months of TB treatment, systems are needed to retain patients in treatment – if they stop treatment too soon, TB can return, potentially in a drug-resistant form, risking their lives and those of others. Social protection payments are important for retention in care and improved treatment outcomes.^13^ Contact investigation – and the provision of preventive treatment for contacts – are additional PHAs that are critical to decrease TB incidence over time.^3,14^

**Table 3.** Characteristics of the TB response that affect how TB should be financed.

Characteristic of TB and the TB response	Consequences for financing
As an airborne infectious disease, TB has high externalities	Market forces will be insufficient to meet the financing needs for TB
TB predominantly affects low-income, vulnerable populations	Relying (either explicitly or implicitly) on out-of-pocket financing for TB care is particularly problematic
Significant TB spread can occur prior to healthcare seeking	Financing for public health actions is needed for: • Active screening in communities and facilities • Engagement of private providers • Contact investigation and preventive treatment
TB can be easily missed even in health facilities	
TB infection is not widely perceived by clients as a threat	
Even in high TB burden countries, active TB disease is rare (compared to issues such as diabetes), but complex and expensive to treat	A large incentive is needed to motivate both diagnosis and correct treatment and support for people with TB Separate financing for public health actions related to adherence support is often also needed

Most health financing dialogue, just like most health financing, focuses on financing for clinical care (i.e., IHAs). However, the PHAs highlighted above require staff time and specific financing to support the staff and their activities. In high TB burden countries, this need is significant enough to require, for example, the majority of TB funding from both the largest bilateral donor in Ethiopia^15^ and a US$400 million loan in India.^16^ These public health needs are often neglected in health financing discussions; they are also among the most donor-dependent parts of current TB programs in many high TB burden countries. Such needs must be central to a TB-specific approach to health financing.

In low-income countries, public sector health financing is driven predominantly by simple supply-side financing in which the government pays directly for the salaries and other operating costs for public sector health facilities (Table 4). In these circumstances, public facility staff may perceive their salaried job as a mix of both IHAs and PHAs, and the direct activity costs related to these PHAs (such as transport costs for outreach) may come from international donors. However, as a country’s economy grows, two changes are commonly seen: donor funds diminish, and domestic financing moves towards demand-side financing, such as through national or social health insurance (SHI). It is important to include TB in SHI schemes and in social protection schemes that cover non-medical costs, which can both reduce TB patient catastrophic costs and provide nutrition that improves TB outcomes.^18,19^ This inclusion provides another important domestic financing source for TB beyond traditional TB-specific budget lines. Within SHI schemes, it is possible to have funding for some PHAs (see section on ‘Strategic purchasing’), but in general SHI schemes are focused on supporting IHAs. In the shift to SHI, the importance of PHAs can be easily neglected, resulting in the under-funding of PHAs.^17^

**Table 4.** Types of financing and their implications for TB programs.

Type of financing	Description	Implication for TB program
Supply side	Government pays directly for the salaries and other operating costs for public sector health facilities; often there is no support for private facilities	Supply-side financing can pay for both PHAs and IHAs. Public facility staff may perceive their salaried job as a mix of both, and/or be encouraged by donor-funded inputs and incentives to make PHAs part of their job. Supply-side financing can also pay for both district-level staff and community health workers with important roles in delivering PHAs, although this may rely significantly on donor funding
Demand side	SHI is used to pay providers – usually in both public and private facilities. Care-seeking patterns of clients, and their demands for services, determine the distribution of money to providers: more clients leads to more money	SHI is designed primarily to pay for IHAs. Regulations may require providers to undertake PHAs but implementation may be incomplete

PHA = public health action; IHA = individual health action; SHI = social health insurance.

A recent cautionary tale comes from Viet Nam, where the inclusion of TB in SHI has led to a ready source of domestic funding for clinical TB care,^20^ but has reduced the focus on domestically financing TB PHAs. Such tension can also be seen in Indonesia^21^ and, in the context of the 2019 Universal Health Care (UHC) Law,^22^ the Philippines. In both the Philippines and Viet Nam, the TB program has been asked to decide whether the TB response should be classified and financed as an individual (clinical) or population-based (public health) effort – whereas the reality is that it is both. As health financing reforms progress, TB programs must underscore the need for strong financing channels for both the clinical and the public health aspects.

## MOBILIZING RESOURCES FOR TB: WHAT COMES FROM WHERE?

The approaches used to advocate for^2^ and monitor^23,24,25^ the mobilization of resources for TB have generally focused on the aggregate levels of funding needed and available. This may be the most sensible approach at the global level – even at the national level, a simple advocacy request for ‘more money’ has a role in mobilizing funding from national parliaments for large centrally funded budget lines such as diagnostics and drugs. However, there is more to domestic resource mobilization than just asking for more money.^26^ A more nuanced analysis and set of decisions around what needs to be funded and from what domestic source is also needed within high TB burden countries. National TB programs (NTPs) need a comprehensive understanding of the full spectrum of existing domestic financing channels, along with an awareness of which channels are most likely to expand over time. This knowledge is crucial to effectively align these funding sources with the diverse components of the TB response.

Dialogue on these topics has been conducted in both Ethiopia^27^ (based on a defined methodology^28^) and Kenya.^29^ The process can include a variety of multisectoral stakeholders (Table 5) and discussion of topics such as:

- Whether domestic funding for each of the various categories of IHA and PHA costs would be more appropriate from one or more levels of the government (national, provincial, or district). Of note, the domestic responsibilities for financing community health workers and public health programs often lie with subnational governments, but those subnational governments may have limited political will, fiscal space, and/or administrative capacity for this role,^30^ and many TB costs may be hidden in integrated primary care programming.
- Whether the financing and procurement of TB commodities (e.g., drugs and diagnostics) should remain under the NTP (as in Indonesia,^19^ even as TB clinical services are paid by SHI) or be folded into SHI payments (as in Viet Nam^18^). Keeping them under the NTP puts the burden on the NTP to supply these commodities directly to individual providers and facilities in both the public and private sectors, which has proven difficult.^31^
- Whether national-level TB program funding can be used to leverage co-financing^28^ by subnational budget holders (e.g., as done in regions of Ethiopia^32^ and counties in Kenya). Co-financing has been implemented for health funding in countries such as India, Indonesia, Kenya and Nigeria: it can raise funds and focus performance on shared goals, but enforcement of co-financing is challenging.^33^
- Whether various TB PHAs should be financed separately from, or as a mandatory bundle with, IHA payments.

**Table 5.** Stakeholders to consult in analyzing current and future domestic financing channels for TB.*

Institutional category	Ethiopia^27,28^	Kenya^29^
Ministry of Health	Strategic Affairs Executive Office [formerly Partnership and Coordination Directorate (PCD)] and the Disease Prevention Lead Executive Office [formerly Disease Prevention and Control Directorate (DPCD)]	Policy and Planning Department
National TB Program	National TB and Leprosy Programme	National Tuberculosis, Leprosy and Lung Disease Program
Subnational authorities	Regional planning and monitoring and evaluation directorate, TB team, grant management unit, resource mobilization directorate, and bureau of finance and economic development. Woreda and zonal health offices and finance offices	County Departments of Health: County Directors for Health; County TB Coordinators; Policy & Planning Officers; M&E Officers County Treasuries: Finance Officers
Ministry of Finance	Federal Ministry of Finance	Ministry of Finance
Health financing advisory groups	Health Financing Technical Working Group	Universal Health Coverage Technical Working Group, National Health Sector Working Group
Health insurance authority	Ethiopia Health Insurance Authority	National Health Insurance Fund
Financial partners	US Agency for International Development (USAID), Global Fund, World Bank	USAID
Technical partners	WHO, German Leprosy and TB Relief Association, KNCV Tuberculosis Foundation, Private Sector Association, REACH Ethiopia, USAID Eliminate TB	WHO, Palladium

* Additional stakeholders are outlined in guidance on multisectoral accountability frameworks.^12^

## IMPLEMENTING THE RESOURCE MOBILIZATION PLAN

Once decisions have been made regarding which domestic funding sources will support specific aspects of the TB response, several additional actions are necessary. First, for advocacy to be successful, TB stakeholders need to be familiar with the budget cycle and the specific advocacy windows and political opportunities within the budget cycle. For example, in Kenya, there are ten steps in the annual county-level planning and budgeting process for TB, but only four of them are a high priority since they involve sub-sectoral (e.g., TB vs other health sub-sector) decisions rather than sectoral (e.g., health vs education) decisions.^34^ Building the necessary budget advocacy capacity may require a proactive training effort.^35^ Resource tracking is another essential input. It generates the current resource numbers, which can then be contrasted with the total funding needed, thus generating a financial gap – which is needed for budget advocacy.^36^

TB financial planners also have to reckon with fragmentation of health financing. Significant levels of devolution (i.e., the transfer of certain powers and resources from national to sub-national governments) are present in many high TB burden countries.^28^ The resultant sub-national financing allows for innovation in response to the local context, but devolution also creates challenges for resource mobilization, pooling and purchasing,^28^ and for appropriate prioritization of TB and implementing a coherent, national TB approach. There are often multiple financial flows from national to sub-national budget units and between different sub-national budget units, and only some may allow coding and earmarking of funds for a specific purpose, such as TB.^37^ When TB codes and earmarks are absent, it can be challenging to determine what resources for TB are truly ‘available’ (or have been spent). Furthermore, the multiple financial flows often come with other limitations (such as spending only on infrastructure or only on certain types of human resources) and become available at different times in the budget cycle. This diversity creates further challenges for implementers attempting to assemble an effective local plan for TB activities.^19,38^

Another tension in domestic resource mobilization for TB exists between having too little and too much ambition. After the limited ambitions of early TB programs,^39^ recent global TB advocacy has prioritized budgetary ambition. The two extremes in ambition in planning are also possible at the national level – and both extremes are worth avoiding. A lack of ambition (such as accepting incomplete coverage of drug resistance testing, inadequate prevention efforts, or active case finding) leads to an underfunded programme that is unlikely to succeed. However, an overly ambitious domestic budget request for TB that lacks reality (e.g., it would take up more than half of the total domestic health budget) can be easily ignored by budgeting authorities.

There is limited documentation to guide national stakeholders through these domestic TB planning and budgeting dynamics. To date, the focus in guidance on developing TB national strategic plans (NSPs)^40^ has been on generating the TB programmatic targets and financial numbers. This guidance does specify that countries should identify the funding source for each intervention in their NSP.^40^ However, for TB NSPs to be more than an international advocacy tool, more practical linkages need to be made to broader subnational and national planning processes (e.g., medium-term expenditure frameworks (MTEFs)) and the constraints of domestic budget cycles.

## STRATEGIC PURCHASING

### Performance-based financing

As noted above, much of health financing in low-income countries involves paying for inputs such as salaries. This has the benefit of simplicity but does not communicate or enforce any particular performance expectations. As economies grow, there tends to be greater use of strategic health purchasing, in which some of the funding allocation is linked to performance metrics and population health needs.^41^ Payment shifts away from inputs and towards outputs related to the quantity and/or quality of services.

There are challenges with such performance-based financing,^42^ as it can result in service providers focusing only on the payment-linked metrics rather than overall quality. Government units may also struggle to undertake the innovation necessary to reach the designated targets due to bureaucratic inflexibilities (e.g., related to hiring or overly centralized budgeting).^42^ However, it may have an important role to play in TB care, where payment metrics could incentivize the provision of public health goods that might otherwise be under-provided. In addition, private sector organizations tend to have a more flexible approach and are able to innovate human resource and governance solutions guided by performance metrics. This is one of the benefits of contracting private organizations (either non-profit or for-profit) using government financing.^43^

## CONTRACTING ORGANIZATIONS FOR PHA SERVICES

Contracting organizations to provide selected TB services allows for complementary skill sets from the public and private sectors, including creative mixtures of both (such as a private implementer running public testing equipment in public labs to extend operating hours). For example, in some countries, non-government organizations (NGOs) may be experienced at organizing community outreach (such as active case-finding), or the engagement and support of large numbers of individual private providers for high quality TB services. NGO contracting can also provide surge support for new initiatives, such as a major push on contact investigation and preventive treatment, particularly in settings where the hiring of additional governmental workers may be bureaucratically challenging.

In high TB burden countries, TB programmes frequently contract organizations, primarily NGOs. However, this is predominantly done through donor funding and contracting arrangements rather than through domestic funding mechanisms subject to local contracting systems and regulations. In many countries, donor-funded NGOs are the backbone of community-based TB programming and there is no exit strategy for these arrangements. Developing the capacity for governments to take on this contracting function is an essential pathway to ensure the sustainability of TB programs in these countries – particularly for the provision of PHA services. One foundation for such a transition is defining packages of TB services that are manageable in complexity, easily costed and measured, and that provide patients with a seamless care pathway and experience. This process has been outlined in detail in India^44^ and for Eastern Europe and Central Asia.^45^

These TB packages differ from the (‘social’) contracting packages assembled for middle-income country HIV programs^46^ in two important respects. First, TB (and potentially HIV) contracting can encompass private organizations that are of various sizes and non-profit and for-profit,^47^ whereas HIV contracting has tended to focus on smaller community-based non-profit organizations that are managed by and provide services to HIV key populations (a service package that government staff may find hard to implement directly). Second, in contrast to HIV, organizations may find it challenging to tap into clinical payments for TB from SHI, even in an enabling environment such as Thailand.^46^ This is because the steps of maintaining clients on long-term HIV treatment are more straightforward than the range of diagnostic and treatment steps (and PHAs) needed for each new TB patient. Thus, although TB and HIV programs benefit from cross-fertilization in efforts to build government and NGO contracting capacity, there are also important distinctions between the two.

There is a lot of guidance on how to operate ongoing contracting,^48^ but relatively little on how to establish a framework for domestic contracting processes.^49,50^ There is no single pathway for countries to follow in establishing health services contracting, but political will (including a clear, country-specific definition of why contracting is a necessary solution) is a foundation for all subsequent steps.^49,50^ In Bangladesh, the multi-year process to establish domestically funded and operated TB contracting has included assessing private sector capacities, government capacities, the political environment and the legal, regulatory and policy environment.^51,52^ Building on such analyses, Bangladesh developed a roadmap to establish TB contracting^53^ with considerable stakeholder engagement.^54^ For government staff, the contracting of organizations for selected parts of the TB response requires new skill sets in managing the contract cycle, including processes for conducting needs assessments (to define what to contract), costing (to determine an appropriate budget for contracting), specifying services and payment metrics (to ensure clarity around expectations on both sides), and ensuring timely payments.

Among high TB burden countries, India provides an instructive example of how to work through these issues, specifically with regard to contracting TB services.^47^ One critical step is to diagnose the contracting challenges,^55^ including those related to ensuring timely submission and payment of invoices^56^ (which, if not solved, may require a source of bridge financing for the implementing organizations^57^). This capacity-building process has been assisted by developing a suite of new tools for conducting needs assessment, tracking pre-contracting steps, defining model bidding and contract documents, standardizing the submission, verification and validation of invoices, tracking payments, and visualizing contract outcomes.^58^ Such optimization is critical since India now implements ∼12% of its TB program via domestically funded contracting.^47,59^

## TB APPROACHES UNDER SHI

Another form of strategic purchasing is the contracting (usually in the context of SHI) of large numbers of individual providers for a broad suite of health services (as compared to the examples above on the selective contracting of individual organizations under narrow, customized terms of reference). The priorities of TB programs in the context of SHI schemes have been previously reviewed (Table 6).^60^ SHI schemes pay providers, and with payment comes the power to incentivize and change the behaviors of providers and patients. Most of this payment and, therefore, most of the incentives are focused on IHAs (diagnosis and treatment; Table 6). But SHI can, at least partially, pay for PHAs. In Taiwan, the payment of TB-related insurance claims was sufficient to cover the cost to the hospitals of hiring TB case managers, who were required by regulation and were responsible for providing many of the PHAs related to TB care.^65,68^ This approach of requiring PHAs (and hiring public health staff as part of the package of reimbursable services) is more tenable where larger organizations or networks are providing TB services.^69^ This is true in Taiwan, which has a hospital-based TB treatment approach because most TB patients are elderly with multiple comorbidities.

**Table 6.** TB financing considerations under SHI.

Possible SHI-related recommendation for TB	Rationale and/or evidence
Preferential enrollment of low-income populations in the SHI scheme	This will maximize the likelihood that clients will already be insured by the time they develop active TB disease, based on the higher prevalence of TB in low-income populations.
Conditional SHI payment: Require TB notification before providers receive an SHI payment for TB-related services	The no-TB notification, no reimbursement policy in Taiwan^61^ and a similar initiative in Indonesia (requiring a TB code in Indonesian hospital TB treatment claims) both resulted in increased notifications. For Indonesia, it was associated with an increase in TB case notification from 54% in 2021 to 74% in 2022, including the identification of 95,571 potentially underreported TB cases in the insurance information system during 2022 alone^62^
SHI payments that are specific to TB	TB-specific payments are needed to draw sufficient attention and prioritization to TB since TB is both rare (compared to issues such as diabetes) and burdensome (due to the expensive diagnostics and long treatment regimen). Relying only on general payment approaches such as capitation can result in increased overall health system costs due to excessive up-referrals^63^
Fee-for-service payment for molecular diagnostics for TB	A fee-for-service payment will incentivize the use of molecular diagnostics for TB; this is beneficial since these tests have a public health benefit and will otherwise tend to be underutilized
Bundled payments for TB treatment (rather than paying for each clinical encounter)	Bundled payments^64^ can focus providers on the achievement of treatment completion, which is what is most important both for patients and for the public health approach. Such bundled payments have been used in a pay-for-performance effort in Taiwan,^65^ and they are being piloted as a way to promote improved TB service provision in primary healthcare in Indonesia^66,67^

SHI = social health insurance.

However, in many high TB burden countries, policies that concentrate TB services in secondary facilities have negative impacts on both patient access and cost.^63^ These countries have more complex decisions to make about who to pay.^66^ For each stage of the TB diagnostic and treatment cascade, it is essential to clearly identify who should be compensated i.e., which cadres and types of organizations at various levels of the healthcare system (across both public and private sectors) will be eligible to provide TB care and receive specific types of TB-related payments. Only after such decisions can there be a clear view of who will provide complementary PHAs for patients under care in these various facilities. In an LMIC setting, where facility types and capabilities are still evolving and patient journeys may be complex, this conversation about where TB diagnosis and treatment ‘belongs’ can be a challenge. Variations in the supply-side readiness of facilities are common, sometimes requiring proactive steps to equip facilities to provide SHI-funded TB services.^70^ For smaller facilities, Indonesia piloted four levels of TB certification for facilities of varying sizes and capabilities. The certification contributed points towards recredentialing facilities for access to SHI payments for TB and increased the number of private primary facilities with the capacity to provide TB services three-fold.^71^ Clarification of PHA responsibilities can be part of this regulatory approach.

If the diagnostic journey is across multiple facility types (e.g., chest X-ray in one location, GeneXpert in another and a diagnosing physician in a third), referral and cost-sharing agreements and mechanisms are needed. This can be aided by the presence of healthcare provider networks (HCPNs) being introduced under the Philippines UHC law, in which care and payments are shared across a network of providers with different capabilities.^72^ For example, the facility accreditation requirements for the recently introduced PhilHealth Konsulta outpatient package require either the onsite presence of staff trained in smear microscopy, or documentation of a service delivery arrangement with another facility with such capabilities.^73^ In theory, PHA responsibilities can also be assigned to this network as a whole rather than solely to the individual facilities. However, if networks do not exist, additional external PHA capacity (e.g., case managers) is needed to ensure a seamless patient journey.

In designing these approaches, financial and regulatory inputs can work well together.^74,75^ Financing provides a carrot to complement the regulatory stick, and the funding can also compensate providers for any cost of adhering to the regulation. However, there is still a risk that TB is either left out or deprioritized in such regulatory and financial schemes. For example, in Cambodia, efforts to align various health facility payments with quality metrics^76–78^ include accreditation standards that lack any requirements specific to TB. Furthermore, TB workers are often deprioritized when performance-based facility income (plus out-of-pocket facility income) is disseminated to staff, as TB is seen as a non-income-generating department. This emphasizes the need to include and recognize TB public health measures, contributions, and outcomes in regulatory approaches and financing schemes, even in clinical facilities.

Finally, finding space in the SHI budget for these public health components is more challenging if the SHI scheme is relatively new or underfunded. Even in countries with larger SHI schemes, additional funding of TB PHAs under the regular health sector budget will likely still be needed for larger-scale community-based and provider-engagement TB activities. These non-SHI public health resources must then be coordinated with the clinical providers receiving the SHI TB payments.

## FUTURE DIRECTIONS

The public health threat of TB has led it to be mainly defined through the lens of vertical national programs. Therefore, it is not easy to build a bridge between the worlds of TB and health financing in high TB burden countries. There are institutional and cultural differences between technical staff in NTPs, the health financing units in the Ministries of Health (MoH), the health staff within the Ministries of Finance (MoF), and the benefit and payment staff in SHI agencies. Overcoming these differences takes leadership and political skill by domestic champions – an under-studied area that would benefit from additional social sciences research. Among these stakeholders, the MoF can be important in highlighting two issues in particular.^4^ First, the MoF is more likely to emphasise the efficiency and outcome gains that could result from a greater shift to contracting and demand-side financing, which can help to involve private organizations and private providers in the provision of TB care.^29,70^ Second, the MoF may be more aware of the potential for cost savings arising from an investment in PHAs. These are important dynamics to capitalize on when considering the way forward for domestic TB financing.

Broad health financing reforms have their own timetable and do not wait for vertical programs such as TB to be ready. However, if TB stakeholders develop skills in areas such as the design of financing strategies, budget advocacy, resource tracking, contracting of organizations, and insurance payment design – and know how to optimise these approaches to meet the unique clinical and public health needs of TB programs – there is a greater likelihood that TB programs can take advantage of both the current health financing landscape and any future financing reforms.

TB programs will require a collection of health financing approaches, often including traditional program management budgets, NGO contracting, SHI payments that incentivize desired outcomes, and social protection payments to patients. Throughout these reforms, it is critical to establish a reliable financing source for PHAs. This dedicated support for PHAs would benefit multiple health areas, but be particularly advantageous for TB programs. Therefore, achieving this outcome may need to be spearheaded by the TB community.
